# Diagnostic performance of target vs. vessel μFR in stable coronary artery disease

**DOI:** 10.1186/s12872-025-04757-x

**Published:** 2025-05-02

**Authors:** Wenhao Huang, Yajun Liu, Qianqian Wang, Hongfeng Jin, Yiming Tang, Jiangting Wang, Xiaowei Liu, Yitao Guo, Chen Ye, Lijiang Tang, Changqing Du

**Affiliations:** 1 Department of Cardiology, Affiliated Hospital of Hangzhou Normal University, Hangzhou, 311321 China; 2 Department of Cardiology, Zhejiang Hospital, Hangzhou, 310013 China

**Keywords:** Coronary physiology, Quantitative Flow Ratio derived from Murray Law, Fractional Flow Reserve, Coronary Artery Disease, Diagnostic Performance

## Abstract

**Background:**

We aim to compare with the diagnostic performance of target-position quantitative flow ratio derived from Murray Law (target-μFR) and vessel quantitative flow ratio derived from Murray Law (vessel-μFR) using the fractional flow reserve (FFR) as reference standard. This study may provide more evidence for the novel clinical usage of target-μFR in the diagnosis of coronary artery disease.

**Methods:**

Six hundreds and fifty-six patients (685 lesions) with known or suspected coronary artery disease were screened for this retrospective analysis between January 2021 to March 2023. A total of 161 patients (190 lesions) underwent quantitative coronary angiography and FFR evaluations. In the final analysis, 137 patients (146 lesions) were included in this study. Both of target-μFR and vessel-μFR were compared the diagnostic performance using the FFR ≤ 0.80 as the reference standard.

**Results:**

Both target-μFR (*R* = 0.84) and vessel-μFR (*R* = 0.83) demonstrated a strong correlation with FFR, and both methods showed great agreement with FFR. The area under the receiver operating characteristic curve was 0.937 for target-μFR and 0.936 for vessel-μFR in predicting FFR ≤ 0.80. FFR ≤ 0.80 were predicted with high sensitivity (86.44%) and specificity (88.51%) using the pre-defined cutt-off of 0.80 for target-μFR. A good diagnostic performance (sensitivity 92.98% and specificity 91.01%) was also demonstrated by vessel-μFR which the pre-defined cutt-off was 0.80.

**Conclusion:**

The target-μFR has the similar diagnostic performance with vessel-μFR. The accuracy of μFR does not seem to be affected by the selection of the measurement point. Both of the virtual models have been validated as computational tools for diagnosing ischemia and are instrumental in aiding clinical decision-making.

## Introduction

The functional evaluation of coronary physiology plays a vital role in guiding diagnosis and treatment strategies in patients with known or suspected coronary artery diseases (CAD). The invasive fractional flow reserve (FFR) has the highest recommendation (class IA) for the evaluation of CAD in guidelines [1, 2]. However, the adoption of FFR in daily clinical practice has been limited because of the invasive of the procedure, requirement of pressure wire, and the administration of hyperemic agents [3–5]. The virtual FFR from coronary artery imaging may increase the utility of FFR assessment in clinical practice. Currently, more attention has been paid in these novel applications of virtual FFR derived from coronary artery imaging, such as quantitative flow ratio (QFR), intravascular ultrasound-derived fractional flow fraction (IVUS-FFR) and coronary computed tomography-derived fractional flow reserve (CT-FFR), without pressure wire and administration of hyperemic agents [6–8].QFR has improved the diagnostic performance for identifying hemodynamically significant coronary stenosis compared with the assessment of coronary lesion based on quantitative coronary angiography (QCA) [9]. Accumulating evidence has proved the QFR could provide more evidence for aid clinical decision-making in CAD [10–13]. QFR derived from Murray Law (μFR) was a novel technique for fast computation of FFR from coronary angiography (CAG) and estimated the pressure drop due to coronary stenosis according to semiautomatic delineation of target vessels and FFR simulation from single-angle [14].

The accuracy of virtual FFR derived from coronary imaging is significantly influenced by the precise location of the measurement within the coronary artery tree. Previous study showed that lesion CT-FFR (defined the value at approximately 2–3 cm distal to the target lesion) could reclassify positive patients defined by the vessel CT-FFR or lowest CT-FFR (defined the value at the distal to the target lesion), and that lesion CT-FFR had higher diagnostic performance than vessel CT-FFR [15]. Recently, more and more studies show that clinicians should not only consider the vessel CT-FFR value when making clinical decisions [16, 17]. The measurement of the μFR was always stay at the vessel level (vessel QFR from Murray Law) in most of current studies, and less of the target-position QFR derived from Murray Law (targer-μFR) was reported. Hence less of researches directly compared diagnostic performance between vessel-μFR and targer-μFR. In summary, we speculate that target-μFR may be superior to the diagnostic performance than the vessel-μFR and may better guide catheterization laboratory revascularization strategies.

In this study, we aim to compare the diagnostic performance of target-μFR and vessel-μFR using the FFR as reference standard. This study holds the potential to furnish additional compelling evidence for the clinical application of μFR in the diagnosis and treatment of CAD.

## Methods

### Study population

A retrospective, single-center, observational study was conducted from January 2021 to March 2023, including consecutive patients with CAD who underlying FFR assessment were eligible for enrollment. The inclusion criteria were as follows: (1) age ≥ 18 years; (2) patients with suspected or known CAD; and (3) at least one lesion with 30–80% diameter stenosis (DS%) based on visual estimation. The exclusion criteria were as follows: (1) angiographic evidence of thrombi-containing lesions; (2) severe valvular heart diseases; (3) left ventricle ejection fraction < 30%; (4) angiographic features limiting μFR computation (eg, left main or ostial right coronary artery ongoing ventricular arrhythmias, and significant and persistent tachycardia); (5) significant foreshortening or vessel overlapping; (6) previous coronary artery bypass grafting; (7) inadequate contrast flush; (8) deep catheter intubation into the lesion precluding complete visualization of stenosis; (9) severely calcified vessels; or (10) inconsistent image format.

The study was conducted in compliance with the Declaration of Helsinki, and was approved by the Ethics Review Committee of Zhejiang Hospital (NO. 2018 (23 K)). The clinical Trial Number is ChiCTR2000034248. Individual informed consent was waived due to the retrospective nature of the study.

### Measurement of physiological indices

The radiographic system Allura Xper FD20/10 (PHILIPS Medical Systems, the Netherlands) was used for the angiographic imaging at a rate of 15 frames/s. The contrast medium was injected at a stable rate of approximately 4 mL/s using a pump. The selection of target lesion was that the diameter stenosis (DS%) was 30–80% based on visual estimation demonstrated by visual estimation. A coronary pressure wire (St. Jude Medical PressureWire™ Aeris, St. Paul, Minnesota, USA) was used to calculate FFR with the pressure sensor positioned at 2–3 cm distal to the most severe target lesion of the coronary artery. Before placement, the pressure wire was calibrated and equalized, and intravenous adenosine triphosphate concentration was 150–180 g/kg/min to induce maximum hyperemia of the coronary microvascular system. Simultaneously, the distal and proximal coronary artery pressures at the pressure sensor (Pd) and coronary ostium (Pa) were recorded. Then the pressure sensor was pulled back to the proximal end to assess or correct pressure drift. The FFR was determined by dividing Pd by Pa. Further analysis was performed at the core laboratory using all ICA and FFR data. Thus, a standardized radiographic system, pressure wire, and software were used, and strict protocols were followed for data collection and analysis to ensure accuracy and reliability [6]. FFR eligibility required stable hemodynamics and operator judgment of lesion suitability for physiological assessment.

### μFR measurement

In this research, the μFR with simultaneous CAG independently used the calculated software (AngioPlus Core, Pulse Medical Imaging Technology Co., Ltd., Shanghai, China). The calculation of μFR, which semiautomatic delineation of vessel contours and FFR simulation from single-angle, was enable by this artificial intelligence-based algorithm. The key frame was first selected from an optimal CAG image which had a sharp lumen contour at the stenosis segment. The vessel contours were automatically delineated, then the reference lumen diameter was reconstructed according to Murray Law of blood flow distribution. The proximal and distal reference lumen diameter could be manually adjusted as needed. And an appropriate manual correction was allowed under certain circumstances, such as stenosis or individual contours with wrong automatic recognition. Finally, both μFR of main and side-branches coronary has been calculated [14]. The vessel-μFR (the pressure drop in entire vessel which the target lesion was located) was measured at the most distal stenosis (> 30% DS) within the vessel [9–13]. Then the target-μFR value was obtain at the position recorded during FFR evaluation procedure (the position was located approximately 2–3 cm distal to the target lesion of the coronary artery). Based on previous studies, we use the μFR ≤ 0.80 (both vessel-μFR and target-μFR) as pre-defined cut-off for predicting ischemia myocardial in our research [18–20]. Vessel-μFR and target-μFR will be only performed on the vessels which underwent FFR evaluation. Both of vessel-μFR and target-μFR were calculated independently by single senior physician who was blinded to FFR value, revascularization status and clinical outcomes. Then a second physician independently verified all calcualtions, blinded to clinical data [21]. All analyses were performed at the core laboratory (Zhejiang Hospital), responsible for image processing and μFR calculations.

### Reproducibility of vessel-μFR and target-μFR

The inter- and intraobserver reliability of vessel-μFR and target-μFR measurements was rigorously assessed through blinded reanalysis of 25 randomly selected cases from the study cohort. Each dataset underwent independent evaluation by both the original analyst (intraobserver analysis) and a second qualified operator (interobserver analysis), following identical standardized protocols. All analyses were performed in blinded regarding vessel-μFR value, target-μFR value, FFR values (if available), and each other’s results to ensure objective assessment.

### Statistical analysis

Continuous and binary variables were presented as mean ± standard deviation (SD) and percentages, respectively. Pearson’s or Spearman’s correlation coefficients were used to quantify the correlations. Bland–Altman plots were used to assess the agreements, which displayed the differences between each pair of measurements versus their mean values with reference lines for the mean difference of all paired measurements. The agreement limits were defined as the mean ± 1.96 SD of the absolute difference. To predict functionally significant stenosis (defined as FFR ≤ 0.80), sensitivity, specificity, and the Youden index (defined as [sensitivity/100] + [specificity/100] − 1) were calculated to compare the diagnostic performance. McNemar’s test was used to compare sensitivity, specificity, and accuracy between target-μFR and vessel-μFR. To assess the area under the curve (AUC) of target-μFR and vessel-μFR, receiver operating characteristic (ROC) curve analysis was performed. Intraobserver and interobserver agreement for assessing vessel-μFR and target-μFR was assessed by Bland–Altman analysis and by means of kappa coefficient. All statistical analyses were performed using MedCalc (version 19.0, MedCalc Software, Ostend, Belgium), and P < 0.05 was defined as statistically significant [6]. The substantial intraobserver agreement (κ > 0.60) suggests methodological robustness for serial assessments.

## Results

### Baseline clinical and lesion characteristics

**Fig. 1:**
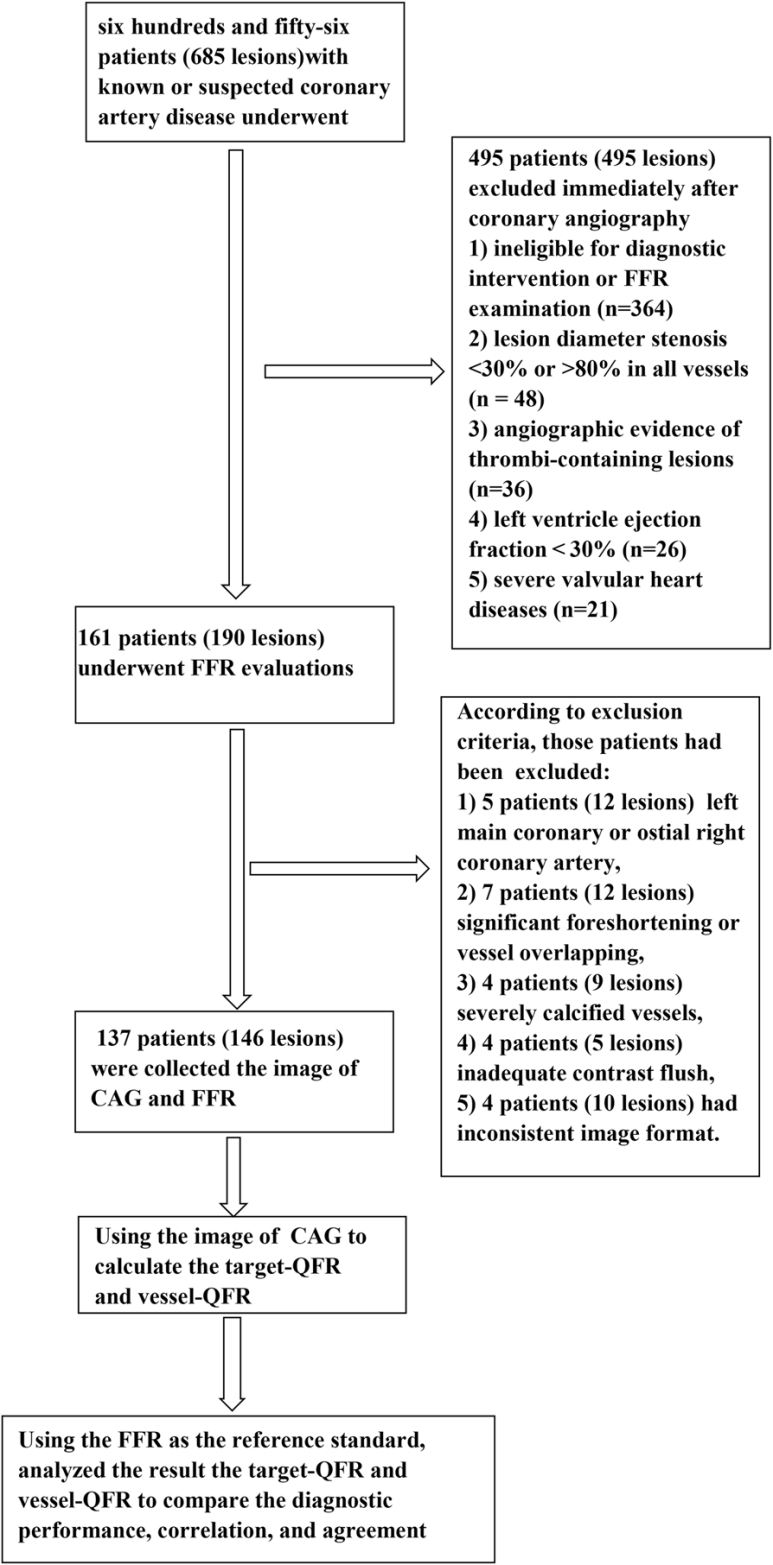
Participant flowchart of the study. FFR: fractional fow reserve, *CAG* coronary angiography, *QCA* quantitative coronary angiography, target-QFR: target-position quantitative flow ratio derived from Murray Law and vessel-QFR: vessel quantitative flow ratio derived from Murray Law

During the research period, six hundreds and fifty-six patients (685 lesions) with known or suspected coronary artery disease were screened for this retrospective analysis between January 2021 to March 2023. A total of 161 patients (190 lesions) underwent CAG and FFR evaluations in our catheter lab. For the excluded patients, five patients (12 lesions) had the lesion in left main coronary or ostial right coronary artery, seven patients (12 lesions) had significant foreshortening or vessel overlapping, four patients (9 lesions) had severely calcified vessels, four patients (5 lesions) had inadequate contrast flush, and four patients (10 lesions) had inconsistent image format. In the fnal analysis, 137 patients (146 lesions) were included in this study (Fig. 1). The mean age was 64.5 ± 10.5 years, and 95 (69.3%) were male. Approximately 44.5% of patients had the history of smoking. The mean left ventricular ejection fraction was 65.8 ± 7.58%. The baseline clinical patient characteristics are listed in Table 1. The target vessels included 92 (63.0%) left anterior descending arteries(LAD), 18 (12.3%) left circumfex arteries(LCX), and 36 (24.7%) right coronary arteries(RCA). The mean values of target-μFR, vessel-μFR, and FFR were 0.83 ± 0.101, 0.83 ± 0.098, and 0.82 ± 0.098, respectively. The baseline lesion characteristics are listed in Table 2.

**Table 1:** Baseline Clinical Patient characteristics

Patient Characteristics	Number of patients (137)
Age,yrs	64.5 ± 10.5
Male	95 (69.3%)
Hypertension	81 (59.1%)
Diabetes mellitus	36 (26.2%)
Hyperlipoidemia	46 (33.5%)
Peripheral artery disease	34 (24.8%)
Cerebrovascular accident history	9 (6.5%)
Smoking history	61 (44.5%)
Drinking history	44 (32.1%)
BMI, kg/m2	23.95 ± 3.18
Family history of CAD	0
Previous CABG	1 (0.7%)
Previous PCI	67 (48.9%)
Previous MI	14 (10.2%)
Clinical presentation	
Stable angina	100(73%)
Unstable angina	37(27%)
Echocardiographic data	
Left ventricle ejection fraction (%)	65.8 ± 7.58
Left ventricle internal dimension (cm)	3.02 ± 0.48
Left ventricle diastolic diameter (cm)	4.76 ± 0.52

Values areMean ± SD or n (%). *BMI* Body mass index, *CAD* coronary artery disease, *CABG* coronary artery bypass grafting, *PCI* percutaneous coronary intervention, *MI* myocardial infarction

**Table 2:** Baseline Lesion Characteristics

Lesion Characteristics Number of vessels (146)	target-μFR	vessel-μFR	*P*
QCA feature			
Diameter stenosis, %	67.64 ± 10.174		-
LAD	92 (63.0%)		-
LCX	18 (12.3%)		-
RCA	36 (24.7%)		-
QFR feature			
Reference vessel diameter, mm	2.94(2.44, 3.29)	2.80(2.34, 3.19)	*P* > 0.05
Minimal lumen diameter, mm	1.61 ± 0.51	1.55 ± 0.47	*P* > 0.05
Diameter stenosis (%)	45.1 ± 10.5	44.2 ± 10.7	*P* > 0.05
Area Stenosis (%)	2.91 ± 0.65	2.78 ± 0.61	*P* > 0.05
Functional indexes			
target-μFR value	0.83 ± 0.101		-
vessel-μFR value	0.83 ± 0.098		-
FFR	0.82 ± 0.098		-
FFR ≤ 0.80	61 (41.5%)		-

Values areMean ± SD, Mean (P25,P75) or n (%). *LAD* lanterior descending coronary artery, *LCX* left circumflex artery, *RCA* right coronary artery. *QCA* quantitative coronary angiography. *Target-μFR* target-position Murray Law based quantitative flow ratio. *vessel-μFR* Vessel quantitative flow ratio derived from Murray Law

### Comparison of the correlations and agreements among target-μFR, vessel-μFR, and invasive-FFR

**Fig. 2:**
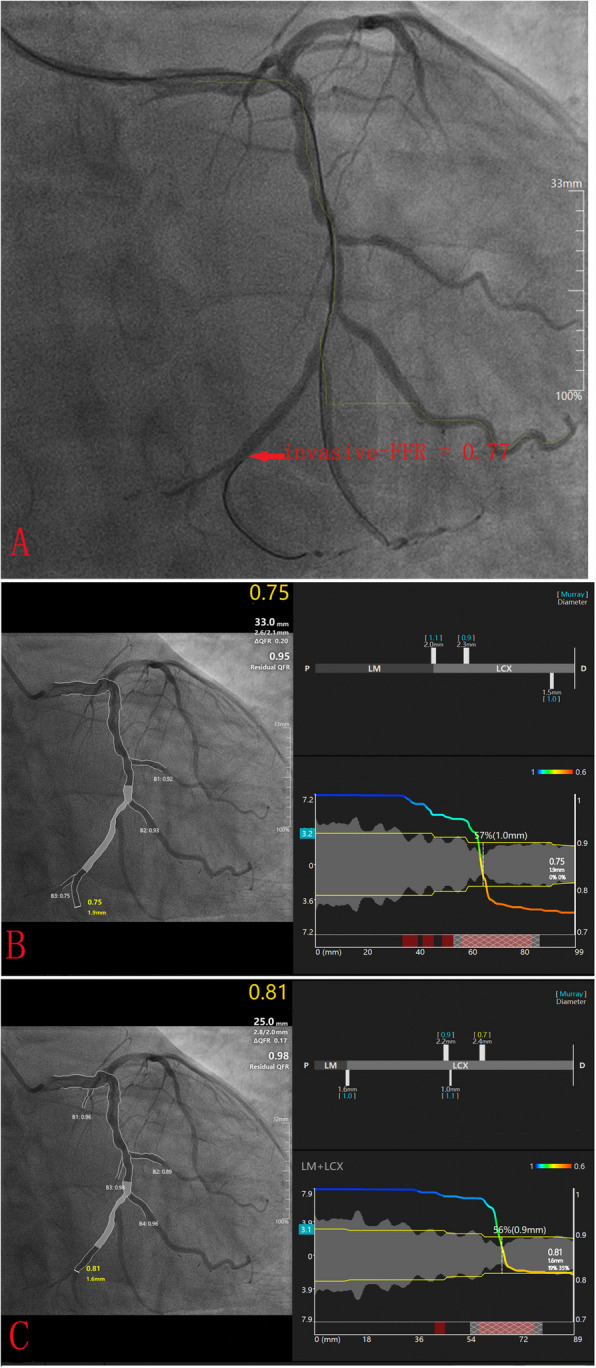
A representative example of target-uFR, vessel-uFR, and FFR measurements. **A** Wire-based Fractional flow reserve (FFR) = 0.77. **B** Vessel quantitative flow ratio derived from Murray Law (vessel-uFR) = 0.75. **C** Target-uFR: target-position. Murray Law based quantitative flow ratio (target-uFR) = 0.81

**Fig. 3:**
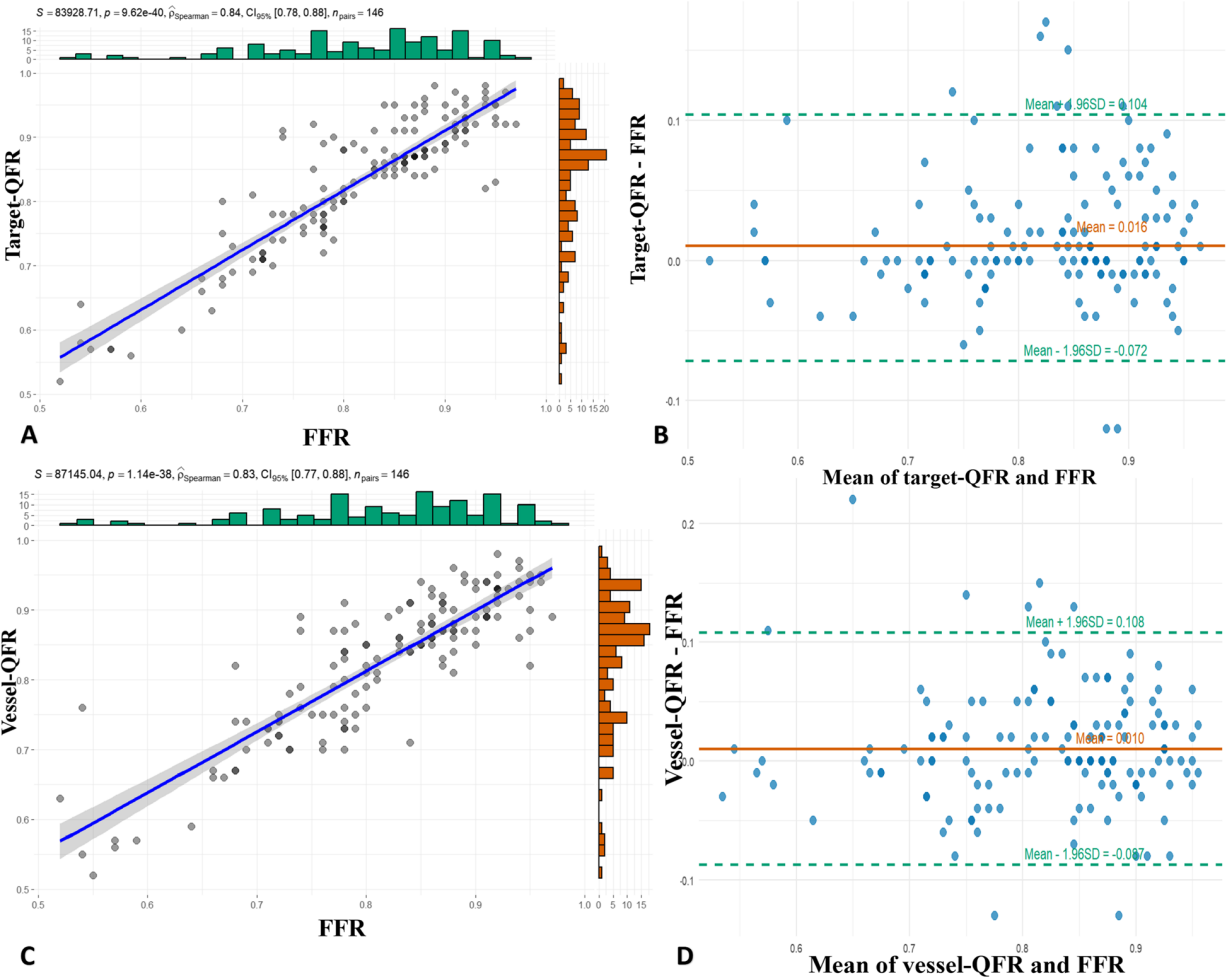
Correlations and agreements among target-QFR, vessel-QFR, and FFR. **A** Correlation between target-QFR and FFR. **B** Agreement between vessel-QFR and FFR. The mean value of target-QFR—FFR = 0.0016, the upper limit of agreement = 0.104, and the lower limit of agreement =—0.072. **C** Correlation between vessel-QFR and FFR. **D** Agreement between vessel-QFR and FFR. Mean value of vessel-QFR—FFR = 0.010, the upper limit of agreement = 0.108, and the lower limit of agreement = 0.087

Figure 2 illustrates a visual representation of target-μFR, vessel-μFR, and FFR measurements. Figure 3 shows the correlation and agreement among these measurements. The results demonstrate that both target-μFR and vessel-μFR had highly correlation with FFR, with R of 0.87 (*P* < 0.05) and 0.90 (*P* < 0.05), respectively. A great agreement is demonstrated by both target-μFR and vessel-μFR with FFR, with similar mean diferences of 0.02 ± 0.045 and 0.01 ± 0.050 respectively. (Fig. 3).

### Diagnostic performance of target-μFR adn vessel-μFR for predicting invasive-FFR ≤ 0.80

**Fig. 4:**
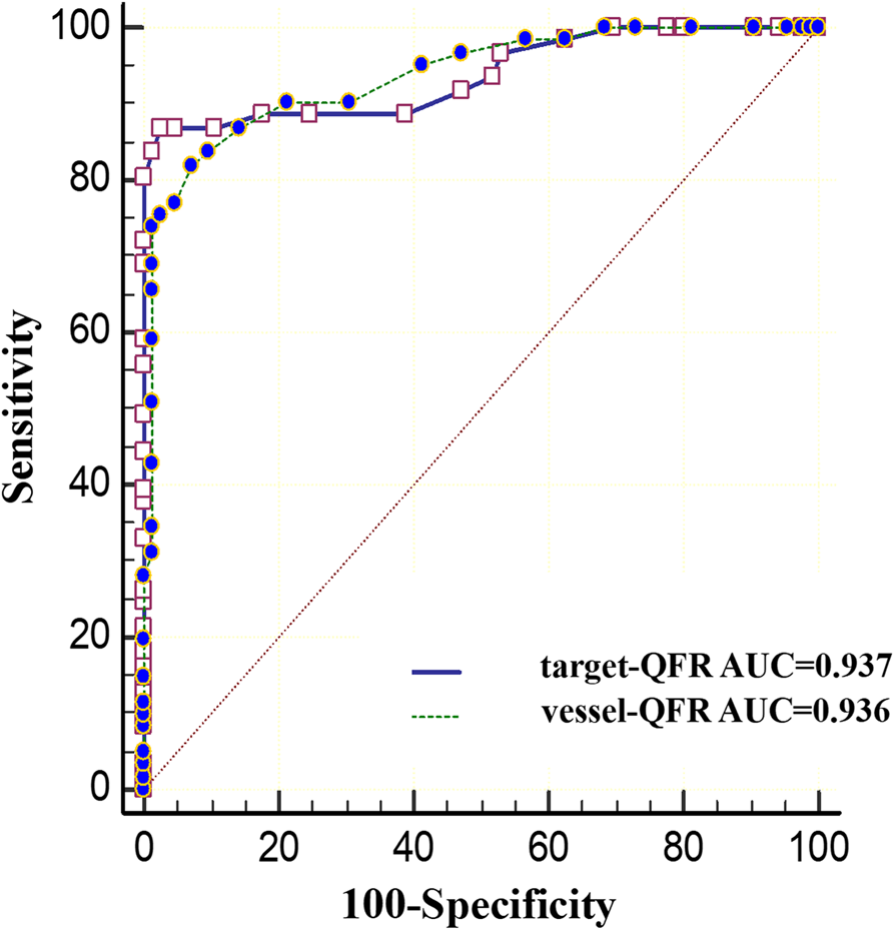
The receiver operating characteristic curves of leison-QFR and vessel-QFR in detecting FFR < 0.80

Figure 4 presents the receiver operating characteristic (ROC) curves for target-μFRand vessel-μFR for FFR ≤ 0.80 predictions. The AUC for target-μFR was similar to that for vessel (0.937 vs. 0.936). The diagnostic performance of target-μFR ≤ 0.80 and vessel-μFR ≤ 0.80 for predicting FFR ≤ 0.80 is presented in Table 3. Among all 146 lesions, using FFR as the reference standard, target-μFR had 53 true positives (TP), 81 true negatives (TN), 4 false positive (FP), and 8 false negative (FN), while vessel-μFR had 46 TP, 83 TN, 2 FP, and 15 FN. Target-μFR had a false discovery rate of 13.11% (positive predict value 86.89%) and a false omission rate of 14.71% (negative predict value 95.29%) compared with FFR. This indicates that 7.02% of stenosis were misclassified using target-μFR compared to FFR. On the other hand, the vessel-μFR also showed a great diagnostic performance, 13.46% were misclassified using vessel-μFR compared to FFR. The diagnostic accuracy of target-μFR ≤ 0.80 for predicting FFR ≤ 0.80 was 91.78%, while that of vessel-μFR was 87.67%. The diagnostic performance indicates that an accurate assessment of coronary stenosis is feasible. What’s more, target-μFR showed numerically higher Youden index values than vessel-μFR (0.840 vs. 0.750, *P* > 0.05).

**Table 3:** Diagnostic performance of target-μFR and vessel-μFR for predicting FFR ≤ 0.80

	vessel-μFR	target-μFR	*P* value
Sensitivity	86.44%(75.02% to 93.96%)	92.98%(83.00% to 98.06%)	> 0.05
Specificity	88.51%(79.876% to 94.348%)	91.01%(3.06% to 96.04%)	> 0.05
Positive Likelihood Ratio	7.52(4.161 to 13.591)	10.34(5.32 to 20.11)	-
Negative Likelihood Ratio	0.15(0.080 to 0.293)	0.08(0.03 to 0.20)	-
Disease prevalence	40.41%(32.38% to 48.84%)	39.04%(31.08% to 47.45%)	-
Positive Predictive Value	83.61%(73.84% to 90.21%)	86.89%(77.31% to 92.80%)	-
Negative Predictive Value	90.59%(83.42% to 94.85%)	95.29%(88.71% to 98.12%)	-
Accuracy	87.67%(81.217% to 92.527%)	91.78%(86.08% to 95.68%)	> 0.05

Value are n(95%confidence interval). Abbreviations as in Table 2

### Reproducibility of vessel-μFR and target-μFR

Repeated μFR measurements (vessel-μFR and target-μFR) were conducted in 25 coronary vessels from a cohort of 25 patients. Interobserver variability for vessel-μFR and target-μFR measurements was 0.02 ± 0.12 and 0.00 ± 0.12, respectively, while intraobserver variability measured 0.02 ± 0.11 and 0.00 ± 0.11. The intraobserver agreement for vessel-μFR yielded a Cohen's kappa coefficient of 0.73 (95% confidence interval [CI], 0.45–1.00; ICC 0.87), compared to an interobserver kappa of 0.68 (95% CI, 0.40–0.95; ICC 0.86). Similarly, target-μFR demonstrated intraobserver and interobserver agreement levels of 0.68 (95% CI, 0.40–0.95; ICC 0.87) and 0.66 (95% CI, 0.35–0.96; ICC 0.83), respectively.

## Discussion

In this study, we compared the diagnostic performance between target-μFR and vessel-μFR using the FFR ≤ 0.80 as reference standard. The primary study findings are summarized as follows: 1) the μFR, a novel physiological assessment methods, estimates the pressure drop due to coronary stenosis according to semiautomatic delineation of target vessels and FFR simulation from single-angle; 2) both target-μFR and vessel-μFR have demonstrated high correlations and great agreements with FFR; 3) the ability of target-μFR defining hemodynamic significant of coronary stenosis was similar to vessel-μFR; 4) the selection of the measurement location has less influence on the accuracy of μFR. Hence, it showed the potential in the coronary imagine and virtual physiological evaluation of CAD. The ability of μFR highlights that by integration of the imaging information in order to enable a comprehensive assessment of the CAD [11–14].

DEFER, FAME and FAME II establish FFR as the"gold standard"of coronary physiology for assessing coronary artery stenosis, treatment plan formulation and evaluation of treatment effect [4, 22, 23]. However, as an invasive method, the application of FFR requires expensive equipment and has potentially procedure-related complications, such as non-fatal myocardial infarction, cerebrovascular accident, and has been limited in clinical practice because of the invasive of the procedure, requirement of pressure wire, the administration of hyperemic agents and so on [3–5, 24]. To solve these limitations and reduce the complications, the QFR had been developed which is a virtual FFR technique derived from coronary angiography. Then μFR as a novel angiographic-based method could enable fast computation of FFR, which provides an avenue for determining the most appropriate therapy for the intermediate lesions.

A large number of clinical studies have confirmed the accuracy of QFR in assessing coronary artery function. In FAVOR Pilot Study, the fix-flow QFR (fQFR), contrast-flow QFR (cQFR) and adenosine-flow QFR (aQFR) was compared with the FFR to evaluate the capability in predicting coronary stenosis. The results confirm that fQFR, cQFR and aQFR had shown the great agreement and diagnostic performance (accuracy 80%, 86%, and 87%) for predicting ischemia myocardial [9]. Then, in the FAVOR II China, QFR demonstrated significantly higher sensitivity and specificity for indentifying hemodynamically significant stenosis compared to QCA (94.6% vs. 62.5%; 91.7% vs. 58.1%). The FAVOR II China also revealed that vessel-level QFR had a high diagnostic accuracy of 93.3% [10]. In a large study of FAVOR II E/J, the good diagnostic performance of QFR assessed the degree of coronary stenosis (accuracy 86.3%, specificity 86.9%, sensitivity 86.5%, AUC 0.92) and evaluated the calculation time of QFR and FFR. Furthermore, the time to complete QFR (5 min) was significantly shorter than the time to complete FFR (7 min) [11]. The FAVOR II China and FAVOR II E/J had proved that the diagnostic accuracy of QFR at both the patients and vessels level was better than QCA in the assessment of the relevance of functional stenoses. Subsequently, Wienemann etal further verified the good diagnostic performance of cQFR was maintained in different clinical subpopulations (including gender, aortic stenosis and atrial fibrillation, etc.) and different anatomical subpopulations (including focal and non-focal lesions, etc.) [25]. In a head-to-head study, QFR showed good agreement with FFR compared with SPECT and PET. Meanwhile, the accuracy of QFR was 88%, 82% for SPECT and 78% for PET [26]. Furthermore, the QFR is the only functional system that has been rigorously evaluated for its clinical value in a randomized clinical trial. In FAVOR III China, after 1-year of follow up, patients randomised to the QFR-guide strategies demonstrated better outcomes driven by fewer myocardial infractions and ischemia-driven revascularisations [27]. Recent findings from the FAVOR III Europe trial highlight the non-inferiority of angiography-derived QFR compared to FFR-guided strategies, supporting the clinical utility of virtual FFR methods likeμFR [28]. Based on those studies, QFR has demonstrated good diagnostic accuracy in detecting myocardial ischemia. Meanwhile, QFR could more conveniently calculate virtual FFR after CAG without any pressure-wire assessments, further providing clinical support for revascularization strategies.

However, the QFR investigations discussed above seldom exhibit target-specific QFR. In previous studies, they demonstrated that the choice of virtual-FFR measurement locations is particular importance when identifying ischemic lesions or guiding treatment strategies [15–17]. Series studies demonstrated that target-specific virtual-FFR, such as target-specific CT-FFR, can reclassify positive patients defined by the vessel-derived FFR value, and that target-specific FFR has higher diagnostic performance than vessel-derived FFR [29–32]. The possible causes of the above phenomenon were as follows: 1) the virtual FFR measurement at far distal segments may overestimate coronary ischemia, 2) these differences between vessel territories in pressure gradients for segments 1–2 cm distal to the stenosis versus far distal segments relate to the larger territory of perfused myocardium, 3) the virtual FFR were only assessed in the main coronary arteries, which may have disregarded the impact of collateral stenosis on myocardial ischemia [6, 16]. Then, Kołtowski Ł et al. analyzed the diagnostic performance of index QFR, vessel QFR (assessment for entire segmented vessel) and lesion QFR (assessment for the target lesion) to identify the best measurement location for optimal accuracy of QFR. The research demonstrated the index QFR value which obtained at the pressure transducer position was the best corresponding QFR model [33]. Although the μFR had been showed great agreement and correlation with standard three-dimensional QFR (R 0.996) [34], the diagnostic performance of target-μFR was similar with the vessel-μFR (*P* > 0.05).

In previous studies, the μFR demonstrated powerful and superior diagnostic performance for target-specific ischemia compared with angiography alone regardless of coronary calcification, and the μFR further reduced the assessment time (67 ± 22 s) [14]. Hence, this paper explores whether target-μFR could further improve the diagnostic ability of myocardial ischemic by comparing the diagnostic performance of target-μFR and vessel-μFR. The diagnostic performance (accuracy 87.67%,sensitivity 86.44%, specificity 88.51%) and AUC (0.936) of vessel-μFR were similar to those reported in previous studies. Then, those indexes of target-μFR (accuracy 91.78%,sensitivity 92.98%, specificity 91.01%), and AUC (0.937) were similar to vessel-μFR and previous research. At the same time, we found the calculation time of target-μFR (1–2 min) and vessel-μFR (1–2 min) was shorter than the QFR reported in previous papers (5 min) [11]. Because of that, the μFR could assess the degree of coronary stenosis which is a time-efficient and accurate method, the visualized anatomic geometry of the coronary artery can provide guidance for subsequent therapeutic regimens. What’s more, based on the U-Net architecture, Murray Law and artificial intelligence, μFR automatically outlines the lumen of the target vessels and their collaterals through artificial intelligence [14]. Hence we suggest the accuracy of μFR have almost less influence by the selection of the remote measurement locations. Based on the difference of the sensitivity, we suggest that the choice of measurement points during the calculation of μFR should be as close as 2–3 cm as possible to the distal end of the culprit vessel which may streamline decision-making by reducing reliance on distal vessel analysis. At the same time, μFR behaved similarly well in sexes and has great diagnostic performance, indicating its potential as a reliable wireless tool for identifying functional ischemia [35].

However, our study had several limitations. First, the study was conducted as a single-center, retrospective analysis with a limited sample size, potentially leading to selection bias despite the inclusion of consecutive patients. Furthermore, the statistical efficiency of the study was compromised by the small number of enrolled patients, which was attributed to the low adoption rate of FFR in clinical practice. Secondly, not all the vessels were interrogated for the enrolled patients. The vessels with diameter stenosis < 30% or > 80% were not assessed because performing physiological assessments in such lesions was unnecessary. Thirdly this is a retrospective analysis in which one-third of the data were excluded because the QFR assessment was not applicable, the study should more likely be viewed as a hypothesis-generating study, and further prospective studies would provide more evidence. The availability of QFR can be improved by requiring careful attention to the projection angle and location of the target lesions in coronary angiography; however, the extent to which this can be improved remains to be assessed. Forth, target-μFR and vessel-μFR computation require automatic reconstruction of 3D anatomical models of coronary vessels, and further studies should be consider that analyze the impact of anatomical features on diagnostic accuracy in target vascular lesions. Fifth, there may be inter-operator differences and previous PCI operation effect in the target-μFR and vessel-μFR calculation process, so further evidences from larger studies are needed. Sixth, the selection of the measurement location depends on the location recorded during the FFR evaluation procedure. Influenced by the real world, some of the measurement locations cannot be accurately positioned at the 2–3 cm distal to the target vessels. Hence, further large-sample, multicenter, prospective, and randomized sthudies are vital to further confirm the feasibility of target-μFR in clinical practice.

## Conclusion

The target-μFR has the similar diagnostic performance with vessel-μFR. The accuracy of μFR does not seem to be affected by the selection of the measurement point. But Target-μFR may streamline decision-making by reducing reliance on distal vessel analysis, particularly in bifurcation lesions. Both of the virtual model could be used as computations tools for diagnosing ischemia and to aid clinical decision-making.

## Data Availability

No datasets were generated or analysed during the current study.
